# Application of multivariate binary logistic regression grouped outlier statistics and geospatial logistic model to identify villages having unusual health-seeking habits for childhood malaria in Malawi

**DOI:** 10.1186/s12936-024-05070-2

**Published:** 2024-08-16

**Authors:** Gracious A. Hamuza, Emmanuel Singogo, Tsirizani M. Kaombe

**Affiliations:** 1 National Statistical Office of Malawi, Zomba, Malawi; 2 University of North Carolina Project, Lilongwe, Malawi; 3 Department of Mathematical Sciences, School of Natural and Applied Sciences, University of Malawi, Zomba, Malawi

**Keywords:** Childhood malaria, Malawi malaria indicator survey data, Caregiver treatment-seeking habit, Mixed-effects logistic regression diagnostics, GeoSpatial statistics, Outlier traditional authorities

## Abstract

**Background:**

Early diagnosis and prompt treatment of malaria in young children are crucial for preventing the serious stages of the disease. If delayed treatment-seeking habits are observed in certain areas, targeted campaigns and interventions can be implemented to improve the situation.

**Methods:**

This study applied multivariate binary logistic regression model diagnostics and geospatial logistic model to identify traditional authorities in Malawi where caregivers have unusual health-seeking behaviour for childhood malaria. The data from the 2021 Malawi Malaria Indicator Survey were analysed using R software version 4.3.0 for regressions and STATA version 17 for data cleaning.

**Results:**

Both models showed significant variability in treatment-seeking habits of caregivers between villages. The mixed-effects logit model residual identified *Vuso Jere*, *Kampingo Sibande*, *Ngabu*, and *Dzoole* as outliers in the model. Despite characteristics that promote late reporting of malaria at clinics, most mothers in these traditional authorities sought treatment within twenty-four hours of the onset of malaria symptoms in their children. On the other hand, the geospatial logit model showed that late seeking of malaria treatment was prevalent in most areas of the country, except a few traditional authorities such as *Mwakaboko*, *Mwenemisuku*, *Mwabulambya*, *Mmbelwa*, *Mwadzama*, *Zulu*, *Amidu*, *Kasisi*, and *Mabuka*.

**Conclusions:**

These findings suggest that using a combination of multivariate regression model residuals and geospatial statistics can help in identifying communities with distinct treatment-seeking patterns for childhood malaria within a population. Health policymakers could benefit from consulting traditional authorities who demonstrated early reporting for care in this study. This could help in understanding the best practices followed by mothers in those areas which can be replicated in regions where seeking care is delayed.

## Background

Patients in sub-Saharan Africa tend to seek medical attention late for malaria treatment, and this behaviour varies significantly across different sub-regions [1–3]. This is due to various factors, including the distance to healthcare facilities, high treatment costs, long waiting times at health facilities, low levels of education, and low household wealth [2–5]. If young children with symptoms of malaria are not treated within 24 h, the disease can quickly progress to dangerous stages [6]. Malaria transmission rates also vary significantly across different areas in sub-Saharan Africa, with some locations being more susceptible to malaria than others [7]. As a result, researchers recommend implementing community-targeted interventions, with some areas receiving control initiatives and others elimination packages, depending on the level of risk [7–10].

To encourage caregivers to promptly seek treatment for childhood malaria, it is important to utilise appropriate statistical tools to identify both areas with desired and undesired habits. This can assist in selecting the right intervention packages for specific areas [11]. Spatial statistics can identify geographic areas with similar or dissimilar health outcomes [12, 13]. Alternatively, grouped outlier statistics can also help detect unique patterns of health outcomes in different areas, after considering relevant risk factors and correlations [14–16]. However, these methods are often used in isolation. This study applies both grouped outlier statistics and geospatial statistics on malaria indicator survey data to identify traditional authorities in Malawi with unusual caregiver treatment-seeking habits for childhood malaria. Employing these two statistical approaches together can help in identifying communities with distinct treatment-seeking behaviour for malaria in children, and contribute to the fight against the disease.

The study’s data and statistical methods, results, discussion, and conclusion are presented in "Methods–Conclusion" sections, respectively.

## Methods

### Data

**Fig. 1:**
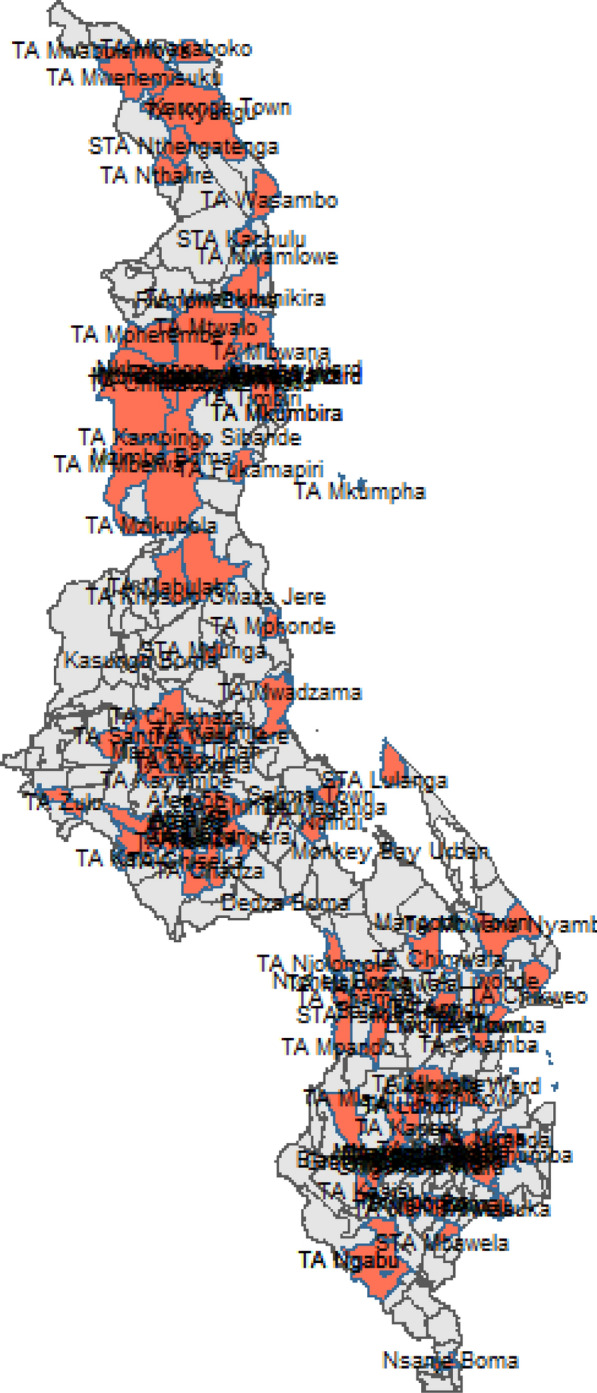
Sampled Traditional Authorities for the 2021 Malawi Malaria Indicator Survey. Source: Researcher

The paper is based on data collected from caregivers who participated in the 2021 Malawi Malaria Indicator Survey, which was conducted between May 3rd and June 30th, 2021 by the National Malaria Control Programme in conjunction with the Measure DHS Programme. The survey randomly selected 150 enumeration areas or clusters from 110 traditional authorities across all districts in the country. Within each cluster, 25 households were sampled, and 3,709 caregivers between the ages of 15 and 49 were included nationwide [17]. This resulted in a sample of 583 under-five children who were included in this research. The children had blood samples taken by laboratory technicians for malaria rapid diagnostic tests or blood smears due to suspected fever two weeks before the survey. The study focused on whether caregivers sought treatment for their children within 24 h if the children showed signs of fever within two weeks of the survey. The study aimed to identify any traditional authorities (TAs) whose treatment-seeking behaviour deviated significantly from the norm, using regression models or spatial statistical methods. The distribution of the sampled TAs is shown in Fig. 1. The regression analysis included factors associated with treatment-seeking habits, such as the sex of the household head, household wealth, caregiver age, religion, ethnicity, education level, region, place of residence, and exposure to malaria messages. [4, 5]. The data were cleaned using STATA software version 14.0. The data are publicly available for users and can be accessed through the link: https://dhsprogram.com/data/dataset/Malawi-MIS-2021.cfm.

### Multivariate binary logistic regression model and estimation

Let $Y_{ij}$ be a binary random variable indicating whether a child’s caregiver *i* in traditional authority *j* sought medical attention for suspected malaria in a child after 24 h of symptom onset. Suppose $y_{ij}$ is the actual observation for the *ij*-th mother. Then $y_{ij}=1$ if the mother presented at the health facility after 24 h, and $y_{ij}=0$ otherwise. If $p_{ij}$ represents the probability that the *ij*-th mother arrives at the facility after 24 h, denoted as $p_{ij} = P(Y_{ij}=1)$, then $Y_{ij}$ follows a multivariate Bernoulli probability distribution with parameter *p*, i.e. $Y\sim Bernoulli(p_{ij})$. Assuming that the parameter $p_{ij}$ remained constant for mothers sampled from the same village, then it could be estimated using the covariates $X_{ij}$ measured on the mother, along with some village-level random covariates $Z_{j}$ that account for the dependence in the observed outcomes from the same village [18] in the following multivariate logistic regresion model setup:

1 $$\begin{aligned} Y_{ij}=p_{ij}(X_{ij},Z_{j})+\epsilon _{ij}, \end{aligned}$$

where

2 $$p_{{ij}} \left( {X_{{ij}} ,Z_{j} } \right) = P\left( {Y_{{ij}} = 1|X_{{ij}} ,Z_{j} } \right) = \frac{{exp\left( {X_{{ij}}^{T} \beta + Z_{j}^{T} b_{j} } \right)}}{{1 + exp\left( {X_{{ij}}^{T} \beta + Z_{j}^{T} b_{j} } \right)}},{\text{ }}$$

and $Y_{ij}'$s have assumed conditional independence in each traditional authority. The explanatory variables $X^{T}_{ijh}=(1,X_{ij1},X_{ij2},...,X_{ijp})$ are regarded as fixed measurements, with $\beta = (\beta _{0},\beta _{1},\beta _{2},...,\beta _{p})^{T}$ being a vector of corresponding coefficients for measuring their effects on $Y_{ij}$. The vector $Z^{T}_{j}=(Z_{1},Z_{2},...,Z_{q})$ represented cluster-level random variables, whose effects were measured by the parameters $b_{j}=(b_{1},b_{2},...,b_{q})^{T}$. The random effects, $b_{j}$’s were assumed to follow a multivariate normal distribution with mean ${\textbf {0}}$ and covariance *D* [19]. The term $\epsilon _{ij}$ was the regression random error for observing each caregiver, which was assumed to have mean of zero.

The model parameters were estimated using the penalised quasi-likelihood technique, where the random effects are considered as nuisance parameters in the likelihood function during the estimation of fixed effects [19, 20]. Assuming that $Z_{j}$ is unity across clusters, where $b_{j} \sim N(0,\sigma ^{2})$, then the penalised quasi-likelihood function for the mixed-effects logistic regression model in Equation (1–2) is given by:

3 $$\begin{aligned} L(\beta ,\sigma ^{2} ) = & \prod\limits_{{j = 1}}^{M} {\prod\limits_{{i = 1}}^{{n_{j} }} e } xp\left[ {Y_{{ij}} log\left( {\frac{{p_{{ij}} \left( {X_{{ij}} ,Z_{j} } \right)}}{{1 - p_{{ij}} \left( {X_{{ij}} ,Z_{j} } \right)}}} \right) + log\left( {1 - p_{{ij}} \left( {X_{{ij}} ,Z_{j} } \right)} \right)} \right] \times \prod\limits_{{j = 1}}^{M} f \left( {b_{j} |\sigma ^{2} } \right) \\ = & exp\left[ {\sum\limits_{{j = 1}}^{M} {\sum\limits_{{i = 1}}^{{n_{j} }} {\left( {Y_{{ij}} \left( {X_{{ij}}^{T} \beta + Z_{j}^{T} b_{j} } \right) - log\left( {1 + exp\left( {X_{{ij}}^{T} \beta + Z_{j}^{T} b_{j} } \right)} \right)} \right)} } } \right] \times \prod\limits_{{j = 1}}^{M} {\left[ {\frac{1}{{\sigma \sqrt {2\pi } }}exp\left( { - \frac{1}{{2\sigma ^{2} }}\sum\limits_{{j = 1}}^{M} {b_{j}^{2} } } \right)} \right]} . \\ \\ \end{aligned}$$

The solutions for the parameters $\beta$ are determined by setting the score vectors obtained from Equation (3) to zero. This process requires iterative numerical methods because the zero equations of the score functions do not have closed forms [19]. The maximum likelihood (ML) estimates ${\hat{\beta }}$ have the usual interpretation, as in univariate logit models. The ${\hat{\beta }}$ represents a change in the logarithm of the odds of seeking care later for malaria that corresponds to a unit change in the covariate *X*. An estimate of the variance of random effects $b_{j}$ shows the amount of variation in the regression intercepts across models for different villages. A variance close to zero shows no variation in the intercepts, while a value larger than zero indicates significant variation in intercept estimates. The estimations were computed using the R package *lme4* [21] in R software version 4.3.0.

### Analysis of outlier traditional authorities to malaria treatment seeking

From the model setup in Equation (1–2), the estimated value for the outcome $Y_{ij}$ can be calculated as:

4 $$\hat{Y}_{{ij}} = \frac{{exp\,\left( {X_{{ij}}^{T} \hat{\beta } + Z_{j}^{T} \hat{b}_{j} } \right)}}{{1 + exp\left( {X_{{ij}}^{T} \hat{\beta } + Z_{j}^{T} \hat{b}_{j} } \right)}}.{\text{ }}$$

Then, the residual for evaluating the quality of fit of each observation in the mixed-effects logistic regression model is determined by the difference between the observed and fitted outcome, given by:

5 $$r_{{ij}} = Y_{{ij}} - \hat{p}_{{ij}} \left( {X_{{ij}} ,Z_{j} } \right) = Y_{{ij}} - \frac{{exp\left( {X_{{ij}}^{T} \hat{\beta } + Z_{j}^{T} \hat{b}_{j} } \right)}}{{1 + exp\left( {X_{{ij}}^{T} \hat{\beta } + Z_{j}^{T} \hat{b}_{j} } \right)}}.{\text{ }}$$

The residuals in Equation (5) exhibit symmetrical values around zero for each group. This symmetry can be useful in identifying single-observation outliers in the mixed-effects logistic regression model. To achieve this, one can graphically plot the residuals in Equation (5) against the fitted values in Equation (4) and identify any unusual observations that exceed a chosen cutoff point [16].

To identify traditional authorities with unusual treatment-seeking practices based on the fitted mixed-effects logistic regression model, a method similar to the one employed in the survival mixed-effects regression model was utilised. The method assumes that the obsersations from different traditional authorities are statistically independent due to the differences in cultural practices, traditions, and habits of the caregivers. This method involves identifying the outlier group by using the ratio of the within-group variance of the residual in Equation (5) to the between-group variance [16]. This is given by:

6 $$\begin{aligned} {\textbf {k}} =\frac{1}{L}\left( \frac{\sum _{i=1}^{n_{1}}(r_{i1}-{\bar{r}}_{1})^{2}}{n_{1}-1},...,\frac{\sum _{i=1}^{n_{M}}(r_{iM}-{\bar{r}}_{M})^{2}}{n_{M}-1}\right) ^{T}. \end{aligned}$$

where $L=\frac{\sum _{j=1}^{M}n_{j}({\bar{r}}_{j}-\bar{{\bar{r}}})^{2}}{M-1}$. The outlier statistic in Equation (6) was used to assess the fit of the mixed-effects logistic regression model for each village. A high value of the statistic indicated a poor fit for traditional authorities, while small values close to zero indicated a good fit [16]. The assessment was graphically performed by creating index plots of the residual in Equation (6) using a cutoff of the 95th percentile of the residual values. These analyses were conducted using the R software version 4.3.0, and the code for it is given in Appendix 1. The results from this analysis were reserved for comparison with those from the geospatial statistical model given in "Spatial logistic regression model, estimation, and detection of areas with similar and dissimilar treatment-seeking habits" section.

### Spatial logistic regression model, estimation, and detection of areas with similar and dissimilar treatment-seeking habits

The 2021 Malawi Malaria Indicator Survey is an observational research that has collected data from various locations, such as clusters and traditional authorities. Therefore, the mixed effects logistic regression model in Equation (1) can be expanded to consider the impact of the underlying Gaussian spatial stochastic process on the behaviour of caregivers in seeking treatment for malaria across the 110 traditional authorities included in the analysis. To identify traditional areas with unusual health-seeking behaviours for childhood malaria in Malawi, the treatment-seeking behaviour variable was classified into a binary outcome: seeking treatment within 24 h (ideal and recommended) and seeking treatment after 24 h (late presentation) from the onset of fever symptoms among the mothers or guardians who participated in the survey.

A geostatistical binomial logistic model [22] was set up to estimate the prevalence of seeking malaria treatment after 24 h at *j*-th location, denoted by $p_{ij}(d(X_{ij}),Z_{j})$, with the resulting response data $Y_{i} \sim Bin(p_{ij})$, for $i = 1,..., n$. Adding a spatially correlated random effect to the mixed effects model in Equation (1), one can obtain the geostatistical binary mixed logistic regression model given by:

7 $$Y_{{ij}} = p_{{ij}} \,\left( {d\left( {X_{{ij}} } \right),S\left( {X_{j} } \right),Z_{j} } \right) + \in _{{ij}} ,{\text{ }}$$

where

8 $$p_{{ij}} \left( {X_{{ij}} ,Z_{j} } \right) = P\,\left( {Y_{{ij}} = 1|X_{{ij}} ,Z_{j} } \right) = \frac{{exp\left( {d\left( {X_{{ij}} } \right)^{T} \beta + S\,\left( {X_{j} } \right) + Z_{j}^{T} b_{j} } \right)}}{{1 + exp\left( {d\left( {X_{{ij}} } \right)^{T} \beta + S\left( {X_{j} } \right) + Z_{j}^{T} b_{j} } \right)}},{\text{ }}$$

and $d(X_{ij})$ is a vector of independent variables associated with the *j*-th location. While *S*(.) is a zero-mean Gaussian process determined from the observed covariates, with variance $\sigma ^{2}$ and correlation function $\rho (X_{j})=corr[S(X_{j}),S(X^{T}_{j})]$. The variable $Z_{j}$ represented the location-specific unmeasured independent normal variates influencing the prevalence of late treatment-seeking habit.

The PrevMap R package was utilised for geostatistical modelling, which involved using a Monte Carlo Markov Chain approximation [23]. It was assumed that the process *S*(.) is isotropic and stationary, meaning that $corr[S(X_{ij}),S(X^{T}_{ij})]=\rho (||X_{j}-X^{T}_{j}||)$, where ||.|| denotes the Euclidean distance. The Matern Covariance function with $\kappa = 0.5$ was used to estimate the covariances for pairs of traditional authorities (TAs), given the separation distance of their centroids. For visualisation, the ggplot and sf packages in R software were used. The 95% confidence intervals for each estimate are reported and the significance of the results are evaluated using p-values at a 5% level. The explanatory variables described in Section 2.1, which were included in the mixed-effects logit model given in Equation (1), were also included in the geospatial model in Equation (8), except for the place of residence and region of stay of the mother, since they were location-specific and had already been accounted for in the geo-coordinates. The R code used for these geospatial analyses is in Appendix 2.

## Results

### Data summary and maximum likelihood estimates from the mixed-effects logit and geospatial logit models

The data summary in Table 1 shows that more than half of the children with fever in the study were brought to the health facility late. The Chi-square test indicated that all the factors examined were associated with the caregiver’s treatment-seeking behaviour, except for four, which had p-values exceeding the 5% significance level. These included the caregiver’s ethnicity, age, place of residence, and exposure to malaria messages. The maximum likelihood estimates for the mixed-effects logistic regression model in Table 1 showed that there was a significant variation in the estimates between traditional authorities. The fixed-effect estimates of the mixed-effects logistic regression model were consistent with those from the geospatial logistic regression model in terms of the direction of the estimates, as seen in Table 1. However, the p-values of fixed effects from the geospatial logit model were generally smaller compared with those from the mixed-effects logit model. Both models showed that the logarithm of odds of reporting a child late for malaria attention at a health facility was lower in households with male heads, middle or rich wealth index, in caregivers aged 24 to 34 years, and those with religions other than Christianity or no religion.In addition, the log odds of late reporting were low in *Tumbuka* or *Yao* tribes, mothers with primary or secondary and above education, those exposed to malaria messages, and those who slept under insecticide-treated nets. On the contrary, the log odds of delayed medical attention were high in women aged 35 to 49 years, Muslims, *Lomwe*, *Ngoni*, or other tribes, and those from urban settings. Without considering the mother’s characteristics, the log odds of delayed reporting for child malaria were expected to increase by a factor of about 2.0 in the study population.

**Table 1:** Distribution of care-seeking behaviour for childhood malaria by characteristics of caregivers and maximum likelihood estimates from the mixed logit and geospatial logit models using the 2021 MMIS data

Factor	Data summary			Mixed logit	Geospatial logit
	n (%)	> 24hr-care (%)	$\chi ^{2}$ pval	Coef (pval)	Coef (pval)
Overall sample/intercept	583 (100)	311 (53.3)		2.35 (0.006)	2.20 (<0.0001)
Sex of household head			0.031		
Female (ref)	478 (82.0)	265 (55.4)			
Male	105 (18.0)	46 (43.8)		− 0.661 (0.009)	− 0.152 (0.456)
Household wealth			0.035		
Poor (ref)	324 (55.6)	187 (57.7)			
Middle	127 (21.8)	65 (51.2)		− 0.407 (0.110)	− 0.059 (0.719)
Rich	132 (22.6)	59 (44.7)		− 0.589 (0.064)	− 1.032 (< 0.0001)
Caregiver age (yrs)			0.28		
15− 24 (ref)	231 (39.6)	124 (53.7)			
25-34	248 (42.5)	125 (50.4)		− 0.207 (0.325)	0.908 (< 0.0001)
35-49	104 (17.8)	62 (59.6)		0.146 (0.603)	2.075 (< 0.0001)
Caregiver religion			0.078		
Christian (ref)	431 (73.9)	228 (52.9)			
Muslim	94 (16.1)	59 (62.8)		1.03 (0.009)	1.11 (<0.0001)
Other religions	55 (9.4)	23 (41.8)		− 0.648 (0.063)	− 2.12 (< 0.0001)
No religion	3 (0.5)	1 (33.3)		− 0.923 (0.503)	− 3.53 (0.062)
Ethnicity			0.52		
Chewa (ref)	196 (33.6)	112 (57.1)			
Tumbuka	89 (15.3)	49 (55.1)		− 0.288 (0.537)	− 0.036 (0.850)
Lomwe	94 (16.1)	42 (44.7)		0.214 (0.579)	− 0.166 (0.495)
Yao	77 (13.2)	40 (51.9)		− 0.277 (0.553)	− 1.454 (<0.0001)
Ngoni	47 (8.1)	26 (55.3)		0.422 (0.301)	0.558 (0.004)
Others	80 (13.7)	42 (52.5)		0.098 (0.816)	− 0.353 (0.054)
Highest Education			0.002		
None (ref)	43 (7.4)	32 (74.4)			
Primary	400 (68.6)	217 (54.3)		− 0.835 (0.047)	− 1.985 (< 0.0001)
Secondary and above	140 (24.0)	62 (44.3)		− 1.137 (0.017)	− 1.678 (< 0.0001)
Exposed to malaria msg			0.25		
No (ref)	18 (3.1)	12 (66.7)			
Yes	565 (96.9)	299 (52.9)		− 0.351 (0.566)	− 0.146 (0.722)
Slept under ITN			0.056		
No (ref)	284 (48.7)	163 (57.4)			
Yes	299 (51.3)	148 (49.5)		− 0.278 (0.153)	− 0.461 (0.002)
Region			0.06		
Northern (ref)	155 (26.6)	88 (56.8)			
Central	209 (35.8)	120 (57.4)		− 0.513 (0.256)	–
Southern	219 (37.6)	103 (47.0)		− 1.114 (0.013)	–
Residential place			0.19		
Rural (ref)	437 (75.0)	240 (54.9)			
Urban	146 (25.0)	71 (48.6)		0.180 (0.558)	–
Random effect var		9		0.569	–

### Outlier villages to child malaria treatment-seeking promptness by mothers based on a mixed-effects logit model

**Fig. 2:**
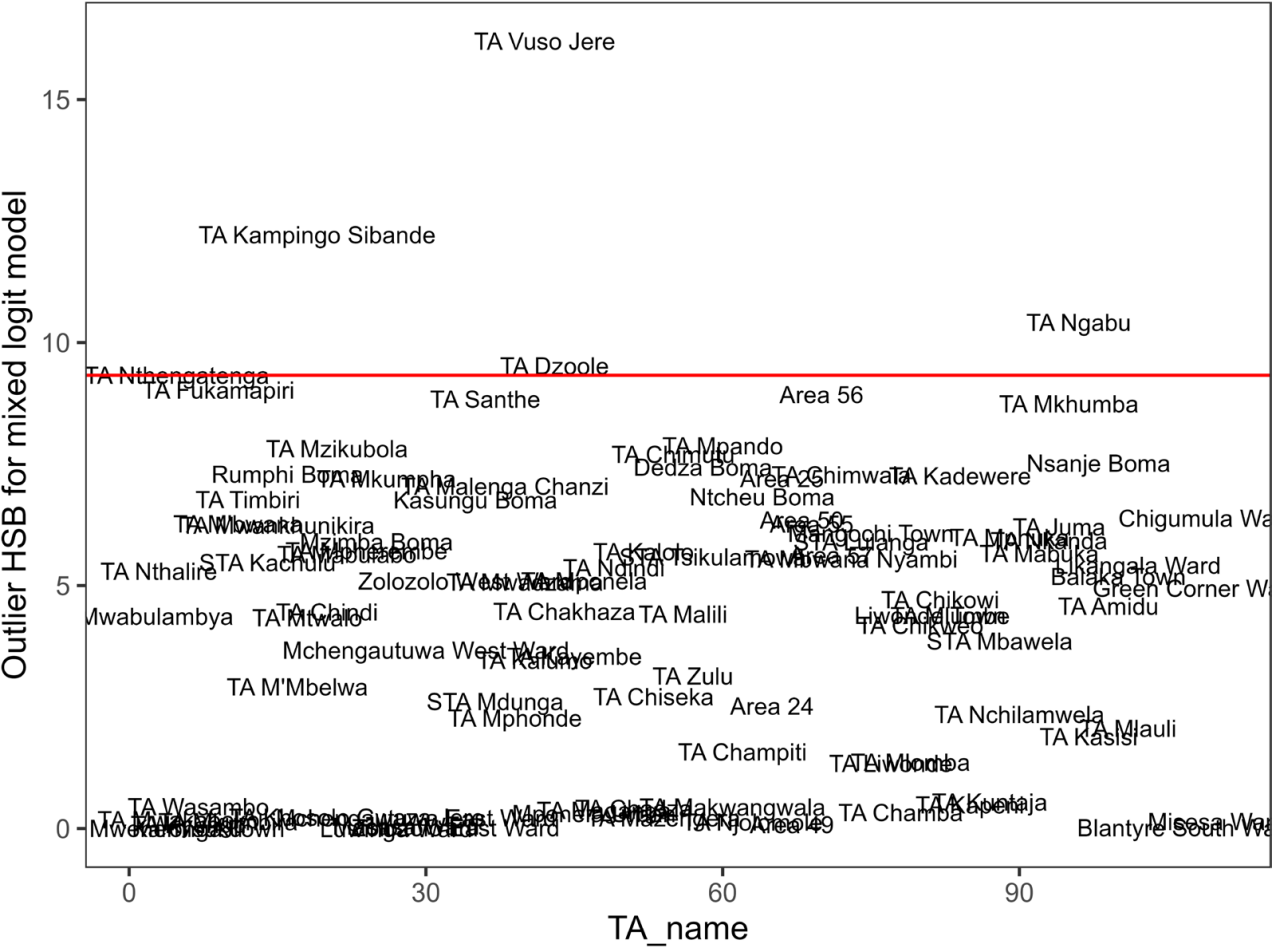
Outlier traditional authorities to health-seeking behaviour for childhood malaria by mothers based on a mixed-effects logistic regression model, 2021 Malawi Malaria indicator survey data. Source: Researcher

The estimates of the multivariate outlier residual in Fig. 2 indicated that mothers in traditional authorities *Vuso Jere*, *Kampingo Sibande*, *Ngabu*, and *Dzoole* had treatment-seeking outcomes that were unusual compared to the other authorities, based on the fitted mixed-effects logit model. Further inspection of the main data set revealed that these outlier authorities had low cases of late reporting for child malaria at clinics, which was opposite to the general trend observed in the study population. Upon examining the data, it was found that the majority of households in these outlier authorities had female heads, were poor, had most caregivers who were not exposed to malaria messages, and a large proportion of children who were not sleeping under insecticide-treated mosquito nets. These factors were linked to an increased risk of delayed reporting for child malaria at health facilities. The detection of these outliers by the applied grouped outlier statistic in this study was therefore justified.

### Geospatial smoothed maps for prevalence of late treatment-seeking behaviour across villages in Malawi

**Fig. 3:**
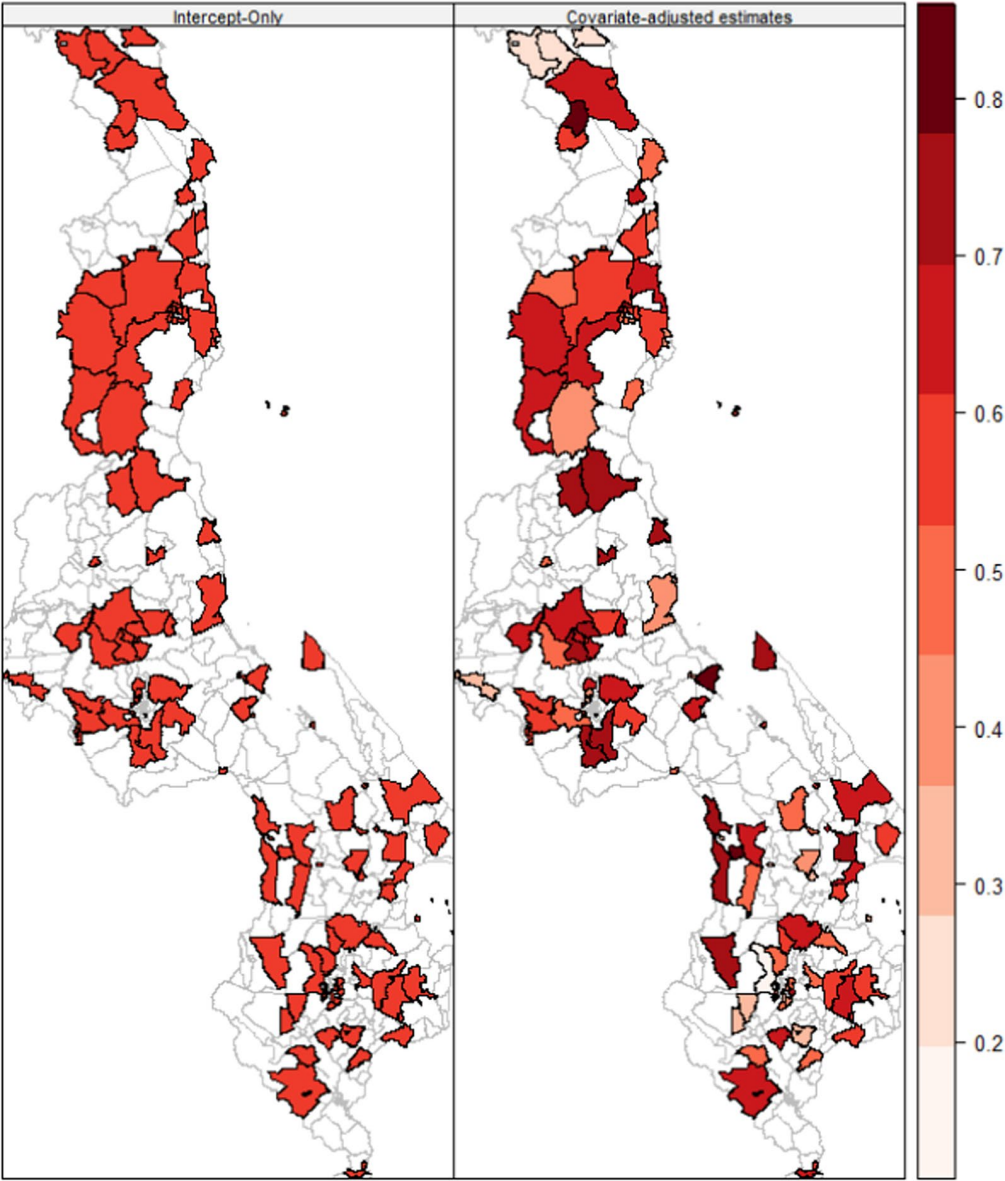
Geospatial smoothed maps showing covariates-adjusted prevalence of late reporting for child malaria treatment by caregivers at health facility per traditional authority based on the 2021 Malawi Malaria indicator survey. Source: Researcher

The results in Fig. 3 are for spatially-smoothed maps of prevalence of late reporting for childhood malaria at health facilities by caregivers. The covariate-adjusted map on the right indicates that the prevalence of late treatment-seeking for childhood malaria varies across the traditional authorities. It shows that more traditional authorities in all regions of the country have a high prevalence of late treatment-seeking habits. Few traditonal authorities had considerably low prevalence of late reporting for malaria at clinics. These are *Mwakaboko*, *Mwenemisuku*, *Mwabulambya*, and *Mmbelwa* in the northern region, *Mwadzama* and *Zulu* in central region, and *Amidu*, *Kasisi*, and *Mabuka* in southern region. Without considering the coverates in the geospatial model, the intercept map on the left showed that prevalence of late reporting for child malaria was high everywhere in the study population.

## Discussion

In this study, national data on child malaria was analysed using mixed-effects logistic regression model diagnostic statistics and geospatial statistical model to identify locations in Malawi with extreme treatment-seeking habits. The results showed that there was significant variation in late reporting for child malaria by caregivers at health facilities between villages. This suggests that accounting for the effect of clustering of outcomes at the village level as well as geographic spacing in the models was necessary [15, 24]. Both models detected a few locations that had a unique pattern of health-seeking behaviour. The mixed-effects logit model identified four traditional authorities that had very low cases of late reporting for malaria treatment at clinics, although the mothers in such locations had characteristics that were associated with high risk of late reporting, according to the model. On the other hand, the geospatial logit model highlights nine locations with the potential for predicted low prevalence of late reporting for malaria, upon accounting for the observed and unobserved covariates, as well as geospatial and environmental correlations in the model. It is worth noting that the extreme areas identified by the mixed-effects logit model were different from those found using the geospatial logit model.

The two approaches used in the study complemented each other in identifying locations with unique patterns of treatment-seeking habits of caregivers. The group outlier statistic for the mixed-effects logit model detected traditional authorities whose data was not well-represented by the model for some reason [16]. In such cases, a follow-up analysis to inspect the data usually helps to understand such outlying trends [25]. When this was performed in this study, it was observed that the characteristics of mothers in the four outlier traditional authorities were associated with an increased risk of late reporting for malaria at health facilities. However, such risk was very low in these locations, which justified why the areas were detected as outliers by the model [14]. On the other hand, the geospatial logit model considered the observed and unobserved factors and geographical spatial characteristics of mothers in a location to predict the prevalence of late reporting for malaria in a particular area [26]. This way, nine locations were observed to have an estimated low prevalence of late treatment-seeking habits, while the rest had high predicted prevalence. In some cases, an analyst may want to determine the impact of grouped outliers on the regression estimates. This can be achieved by using influence measures for the mixed-effects model [15, 25]. To analyse the correct classification of success probabilities in the binary logistic regression model, one can use receiver operating characteristic (ROC) curves and area under the curve (AUC) analyses [27–29]. However, these analyses were not performed in the present study. The objective of the paper was to detect outliers and unusual caregivers at the group level using villages as units of analysis and understand their characteristics. This information can help health policymakers design appropriate interventions for such groups.

Both models have identified similar risk factors associated with late reporting of under-five malaria cases at clinics. The risk is low for households headed by males, wealthier families, mothers with at least primary education, those aged 24 to 34 years, *Tumbuka* or *Yao* tribes, mothers who have been exposed to malaria media messages, those who slept under insecticide-treated nets, and those with religions other than Christianity. On the other hand, the risk is high for mothers aged 35 to 49 years, Muslim caregivers, *Lomwe*, *Ngoni*, and other tribes than *Chewa*. These findings are consistent with previously published studies that have analysed factors associated with delayed presentation for malaria at clinics. The current study corroborates with the significant variables reported in previous studies [3, 30]. However, the estimates from the geospatial logit model produced smaller p-values compared to the mixed-effects logit model. This is because of the different parameter estimation techniques used in each model. The maximum likelihood estimation technique used in the mixed-effect logit model is known to produce larger standard errors compared to the nonparametric Monte Carlo Markov Chain estimation used in the geospatial logit model [31]. Furthermore, the maximum likelihood estimation technique, followed by numerical solutions approximations, is known to be sensitive to outliers in the model [31, 32]. Therefore, this estimation technique was crucial for the detection of the outliers in the model. However, nonparametric estimation methods are considered to be more robust and less vulnerable to outliers in the model [33].

In the event that a researcher aims to identify and confirm the risk factors of delayed reporting for childhood malaria at health facilities, other alternative models that can be used include the multivariate survival model [16] and the multivariate modified Poisson model [34]. A multivariate survival regression can estimate the significant factors associated with the duration of reporting for malaria at a clinic from the onset of fever symptoms by characterizing the hazard of reporting late, while accounting for dependences in the data [15]. Whereas the multivariate modified Poisson model could estimate the relative risk of delayed reporting considering two levels of a covariate taking into account the clustering of the data [35]. When applied to the same data, the binary logistic regression model using the censoring status outcome as the response variable, the censored survival regression, and the modified Poisson model yield similar estimates [16, 34, 36]. The study did not utilise all three models for this reason, as the goal of the paper was to identify the villages/provinces with outlying and unusual health-seeking behaviour, beyond fitting the model to the data and estimating the risk factors.

The MMIS data analysed in this study had some limitations. For instance, caregivers provided information during interviews about how long it took them to seek medical attention for their child’s fever in the two weeks prior to the survey. This introduces the potential for recall bias. Therefore, we recommend that future research should include statistical methods to address recall bias in the multivariate logistic and spatial models used in this study [37, 38]. Moreover, the primary sampling unit for the data was the household. Nevertheless, this study’s analysis concentrated on the timeliness of care for the children rather than for households. This may introduce biases in the estimation. Hence, future research could consider employing advanced sample weighting techniques along with statistical methods to address this issue [36].

## Conclusion

This study aimed to identify villages in Malawi with unusual treatment-seeking behaviour for childhood malaria by using a combination of mixed-effects logistic regression diagnostics and a geospatial statistical model. The results showed that there was a significant variation in the timeliness of seeking care for malaria between villages. The mixed-effects logit model outlier residual identified four traditional authorities, while the geospatial logit model identified nine, as having a unique pattern of treatment-seeking behaviour. The mixed-effects logit model identified the areas where there were very few cases of delayed presentation for malaria at health facilities, even though caregivers in those villages were at a higher risk of delayed reporting. On the other hand, the geospatial logit model identified the areas with a low predicted prevalence of delayed reporting for malaria, taking into account the measured, unmeasured, and geographical spacing factors in the model.

The fixed-effect estimates of the included factors were consistent between the two models. Using either method, the risk of delayed presentation of childhood malaria at health facilities was low in economically stable, male-headed households, mothers with primary education and above, caregivers aged 24 to 34 years, *Tumbuka* or *Yao* tribes, mothers exposed to malaria media messages, those who slept under insecticide-treated nets, and those with religions other than Christianity. However, the risk was high in mothers aged 35–49 years, Muslims, *Lomwe*, *Ngoni*, and other tribes than *Chewa*. Nonetheless, the regression estimates found using the geospatial logit model had smaller p-values than those from the mixed-effects logit model.

The study found that many caregivers in Malawi tend to report child malaria cases late at clinics. However, there are a few traditional authorities that have better practices in this regard. The study recommends that health policymakers learn from these outlier traditional authorities and implement their best practices in other villages to improve the timely seeking of care for child malaria. Additionally, using combined grouped outlier detection methods for mixed-effects logit model and geospatial logit model is suggested to analyse villages with extreme health-seeking behaviour for childhood malaria or other binary health outcomes in a population. This can also be done using panel malaria data to observe the detected outlier villages’ patterns over time. Finally, a follow-up qualitative study is recommended to investigate the practices and traditions of mothers and caregivers in the identified outlier areas with low prevalence of delayed treatment-seeking habits for child malaria as a potential area for future research.

### Supplementary Information

Supplementary Material 1.

Supplementary Material 2.

## Data Availability

The 2021 MIS data are publicly and freely available for users at https://dhsprogram.com/data/dataset/Malawi-MIS-2021.cfm.
