# Supplementary material for: Application of multivariate binary logistic regression grouped outlier statistics and geospatial logistic model to identify villages having unusual health-seeking habits for childhood malaria in Malawi
Source: Malar J. 2024 Aug 16;23:246. doi: 10.1186/s12936-024-05070-2 (PMC11328507; doi:10.1186/s12936-024-05070-2)
Supplement: Supplementary file 1 — Supplementary Material 1. [file 12936_2024_5070_MOESM1_ESM.pdf]

1 Appendix 2: R code used to fit geospatial logit model for 2021 MMIS data cleaning

```
#A. R code for geospatial logit model
library(PrevMap)#modelling
library(geoR) #reading shape files
library(RColorBrewer) #for map colors
library(readstata13)
library(sf)
library(ggplot2)
library(RColorBrewer)

setwd("") #set your working directory

## read data
hsb_dat <- read.dta13("MICS2021III.dta")
ta_table_code <- data.frame(cbind(table(hsb_dat$tacode,hsb_dat$HCSB)))
names(ta_table_code) <- c("Over_24hrs","within_24hrs")
ta_table_code$total_childs <- ta_table_code$Over_24hrs + ta_table_code$
    ↪ within_24hrs
ta_table_code$tacode <- row.names(ta_table_code)

hsb_mics <- read.csv("HSB_mics.csv",stringsAsFactors = T)
hsb_mics$select <- 1 #dummy variable
names(hsb_mics)

#load Malawi districts shape file
mw_dist <- st_read("mwi_admbnda_adm2.shp")
plot(mw_dist[2])
mw_dist <- as(mw_dist,"Spatial")
plot(mw_dist)

#Load Malawi TAs
mw_tas <- st_read("mwi_admbnda_adm3.shp")
mw_tas1 <- as(mw_tas,"Spatial")
plot(mw_tas1)

mw_tas2 <- spTransform(mw_tas1,CRS("+proj=longlat␣+datum=WGS84"))

# Malawi TA centroids
centd <- data.frame(mw_tas2@polygons)
dim(centd) #this should be equal to number of TAs you have

names(centd) <- c("long","lat")

# Add you data to the shape file
hsb_mics_dat <- merge(x=mw_tas,y=hsb_mics,by.x="ADM3_EN",by.y="ta_name",
    ↪ all.x=T)
dim(hsb_mics_dat)
names(hsb_mics_dat)

hsb_mics_datf<- merge(x=hsb_mics_dat,y=ta_table_code,by="tacode",all.x=T)
```

```
dim(hsb_mics_datf)
names(hsb_mics_datf)

hsb_mics_datf$tacode <- as.factor(hsb_mics_datf$tacode)
hsb_mics_datf <- subset(hsb_mics_datf,!is.na(total_childs))

#Plot selected Tas
spplot(hsb_mics_datf,"select",sp.layout=list(mw_tas2,col="grey"),
colorkey=FALSE)

hsb_mics_datf2 <- as(hsb_mics_datf,"sf")

hsb_mics_datf2$TA_numbering <- 1:length(hsb_mics_datf2$ADM3_EN)

TA_map <- ggplot() +geom_sf(data=mw_tas)+
geom_sf(data=hsb_mics_datf2, aes(col=select),
fill="coral1")+ geom_text(data = hsb_mics_datf2, aes(x =avg_longitude , y
    ↪  = avg_latitude, label =ADM3_EN ), size = 2)+
theme(axis.text.x=element_blank(), axis.ticks.x=element_blank(), axis.
    ↪ text.y=element_blank(), axis.ticks.y=element_blank() ) + theme(
    ↪ plot.title = element_text(color="black",size=7, face="italic"),
    ↪ axis.title.x = element_blank(), axis.title.y = element_blank()) +
    ↪ theme(panel.background = element_rect(fill="white",colour = "white
    ↪ "), panel.grid.major = element_blank(), panel.grid.minor = element
    ↪ _blank(), panel.border = element_blank()) + theme(legend.position=
    ↪ "none")

x11()
TA_map
save(TA_map,file="TA_map.RDa")

#Likelihood-based analysis for setting parameters of the importance
    ↪ sampling distribution

#TTI logit functions

hsblogit <- log((hsb_mics_datf$Over_24hrs+0.5)/(hsb_mics_datf$total_
    ↪ childs - hsb_mics_datf$Over_24hrs+0.5))

#TTI data frames
hsbdf <- data.frame(hsblogit=hsblogit,coord1=centd[,1],coord2=centd[,2])

#find empirical variogram parameter estimates
hsbglm <- glm(cbind(Over_24hrs,total_childs)~propMale_hh+middle_wmn +
    ↪ older_wmn+propMuslims +   Other_rel+No_relign + Tumbuka + Lomwe +
    ↪ Yao + Ngoni + Other_tribe + Exp_to_media + Primary + Sec_above +
    ↪ middle_fam + rich_fam + slept_underITN,
              family=binomial,data=hsb_mics_datf)

vari <- variog(coords=centd,data=hsbdf$hsblogit,uvec=seq(0,300000,len=20)
    ↪ )
```

```
vari.fit <- variofit(vari,ini.cov.pars=c(0.5,1000),cov.model="matern",
            fix.nugget=FALSE,nugget=.01,fix.kappa=TRUE,kappa=0.5)

par0 <- c(coef(hsbglm),vari.fit$cov.pars,vari.fit$nugget)

c.mcmc <- control.mcmc.MCML(n.sim=10000,burnin=2000,thin=8,
            h=(1.65)/(nlevels(hsb_mics_datf$tacode)-1)^(1/6))

#Obtaining Intercept-Only Estimates
set.seed(1357)
hsbMCML1_0 <- binomial.logistic.MCML(formula=hsb_mics_datf$Over_24hrs~1,
            units.m=~hsb_mics_datf$total_childs,
            par0=par0,coords=~hsbdf$coord1+hsbdf$coord2,data=hsb_mics_
                ↪ datf,
            control.mcmc=c.mcmc,kappa=0.5,
            start.cov.pars=c(par0[3],par0[4]/par0[2]))

#repeat using new estimates
par0 <- coef(hsbMCML1_0)
set.seed(1357)
hsbMCML2_0 <-binomial.logistic.MCML(formula=hsb_mics_datf$Over_24hrs~1,
            units.m=~hsb_mics_datf$total_childs,
            par0=par0,coords=~hsbdf$coord1+hsbdf$coord2,data=hsb_mics_
                ↪ datf,
            control.mcmc=c.mcmc,kappa=0.5,
            start.cov.pars=c(par0[3],par0[4]/par0[2]))

par0 <- coef(hsbMCML2_0)
c.mcmc <- control.mcmc.MCML(n.sim=300000,burnin=5000,thin=8,
h=(1.65)/(nlevels(hsb_mics_datf$tacode)-1)^(1/6))
set.seed(1357)

hsbMCML3_0 <- binomial.logistic.MCML(formula=hsb_mics_datf$Over_24hrs~1,
            units.m=~hsb_mics_datf$total_childs,
            par0=par0,coords=~hsbdf$coord1+hsbdf$coord2,data=hsb_mics_
                ↪ datf,
            control.mcmc=c.mcmc,kappa=0.5,
            start.cov.pars=c(par0[3],par0[4]/par0[2]))

summary(hsbMCML3_0)

hsbpred.MCML_0<- spatial.pred.binomial.MCML(hsbMCML3_0,centd,control.mcmc
    ↪ =c.mcmc, type="marginal",scale.predictions="prevalence",
      standard.errors=TRUE)

hsb_mics_datf@data$estimates_nocovars <- hsbpred.MCML_0$prevalence$
    ↪ predictions

#Obtaining covariate-Adjusted Estimates
set.seed(1357)
hsbMCML1 <- binomial.logistic.MCML(formula=hsb_mics_datf$Over_24hrs~
    ↪ propMale_hh + middle_wmn + older_wmn + propMuslims +
```

```
                Other_rel + No_religн + Tumbuka + Lomwe + Yao + Ngoni +
                    ↪ Other_tribe + Exp_to_media + Primary +
                Sec_above + middle_fam + rich_fam + slept_underITN,
                units.m=~hsb_mics_datf$total_childs,
                par0=par0,coords=~hsbdf$coord1+hsbdf$coord2,data=hsb_mics_
                    ↪ datf,
                control.mcmc=c.mcmc,kappa=0.5,
                start.cov.pars=c(par0[3],par0[4]/par0[2]))

#repeat using new estimates
par0 <- coef(hsbMCML1)
set.seed(1357)
hsbMCML2 <- binomial.logistic.MCML(formula=hsb_mics_datf$Over_24hrs~
    ↪ propMale_hh + middle_wmn + older_wmn + propMuslims +
                Other_rel + No_religн + Tumbuka + Lomwe + Yao + Ngoni +
                    ↪ Other_tribe + Exp_to_media + Primary +
                Sec_above + middle_fam + rich_fam + slept_underITN,
                units.m=~hsb_mics_datf$total_childs,
                par0=par0,coords=~hsbdf$coord1+hsbdf$coord2,data=hsb_mics_
                    ↪ datf,
                control.mcmc=c.mcmc,kappa=0.5,
                start.cov.pars=c(par0[3],par0[4]/par0[2]))

par0 <- coef(hsbMCML2)
c.mcmc <- control.mcmc.MCML(n.sim=300000,burnin=5000,thin=8,
                h=(1.65)/(nlevels(hsb_mics_datf$tacode)-1)^(1/6))
set.seed(1357)

hsbMCML3 <- binomial.logistic.MCML(formula=hsb_mics_datf$Over_24hrs~
    ↪ propMale_hh + middle_wmn + older_wmn + propMuslims +
                Other_rel + No_religн + Tumbuka + Lomwe + Yao + Ngoni +
                    ↪ Other_tribe + Exp_to_media + Primary +
                Sec_above + middle_fam + rich_fam + slept_underITN,
                units.m=~hsb_mics_datf$total_childs,
                par0=par0,coords=~hsbdf$coord1 + hsbdf$coord2,data=hsb_mics_
                    ↪ datf,
                control.mcmc=c.mcmc,kappa=0.5,
                start.cov.pars=c(par0[3],par0[4]/par0[2]))

summary(hsbMCML3)

hsbpred.MCML_covs<- spatial.pred.binomial.MCML(hsbMCML3,centd,control.
    ↪ mcmc=c.mcmc,
                predictors=hsb_mics_datf@data[,c(22:38)],
                type="marginal",scale.predictions="prevalence",
                standard.errors=TRUE)

#Plot Maps
load("hsb_mics_datf.RDa")
class(hsb_mics_datf)
names(hsb_mics_datf)
dim(hsb_mics_datf)
```

```
hsb_mics_datf@data$hsbmodel_nocovars <- hsbpred.MCML$prevalence$
    ↪ predictions
hsb_mics_datf@data$hsbmodel_covars <- hsbpred.MCML_covs$prevalence$
    ↪ predictions

hsb_mics_sf <- as(hsb_mics_datf,"sf")
names(hsb_mics_sf)

hsbmap2 <- spplot(hsb_mics_datf,c("hsbmodel_nocovars","hsbmodel_covars"),
             names.attr=c("Intercept-Only","Covariate-adjusted␣estimates"
                 ↪ ),as.table=TRUE,
             col.regions=brewer.pal(9,"Reds"),cuts=8,
             sp.layout=list(mw_tas2,
             col="grey"),par.strip.text = list(cex = .7))
x11()
hsbmap2
```
