# Supplementary material for: Application of multivariate binary logistic regression grouped outlier statistics and geospatial logistic model to identify villages having unusual health-seeking habits for childhood malaria in Malawi
Source: Malar J. 2024 Aug 16;23:246. doi: 10.1186/s12936-024-05070-2 (PMC11328507; doi:10.1186/s12936-024-05070-2)
Supplement: Supplementary file 2 — Supplementary Material 2. [file 12936_2024_5070_MOESM2_ESM.pdf]

1 Appendix 1: R code used to fit mixed-effects logistic regression model and compute post-estimation statistics and STATA code for 2021 MMIS data cleaning

```
#A. STATA Data cleaning code for MMIS Data

use "C:\Users\Hp\Desktop\Mixed_Models\Pepa_working_Datasets\working_
    ↪ data_2021_020124.dta", clear

rename head_sex hh_sex
recode hh_sex (1 = 0) (0 = 1)
lab def hh_sex 0 "female" 1 "male"
lab val hh_sex hh_sex
ta hh_sex
ta hh_sex [aw = hhweight]

recode w103 (15/24 = 0) (25/34 = 1) (35/49 = 2), gen(Age_grps)
lab def Age_grps 0 "15-24" 1 "25-34" 2 "35-49"
lab val Age_grps Age_grps
gen young_women = .
replace young_women = 1 if Age_grps == 0
replace young_women = 0 if Age_grps == 1 | Age_grps == 2
gen middle_women = .
replace middle_women = 1 if Age_grps == 1
replace middle_women = 0 if Age_grps == 0 | Age_grps == 2
gen older_women = .
replace older_women = 1 if Age_grps == 2
replace older_women = 0 if Age_grps == 0 | Age_grps == 1

recode w106 (0 = 0) (1/8 = 1) (9/30 = 2), gen(Educ_Levels)
lab def Educ_Levels 0 "No␣Education" 1 "Primary␣School" 2 "Secondary
    ↪ ␣and␣above"
lab val Educ_Levels Educ_Levels
gen Primary_sch = .
replace Primary_sch = 1 if Educ_Levels == 1
replace Primary_sch = 0 if Educ_Levels == 2
gen Sec_and_above = .
replace Sec_and_above = 1 if Educ_Levels == 2
replace Sec_and_above = 0 if Educ_Levels == 1

recode w410 (0/1=1) (2/14=0) (missing = 0) if w404== 1 , gen(HCSB)
lab var HCSB "Days␣taken␣before␣treatment␣was␣sought"
lab def HCSB 1 "within␣24␣hrs" 0 "Over␣24␣hrs"
lab val HCSB HCSB

lab def residence 1 "Urban" 0 "Rural"
lab val residence residence

recode slept_under_net (0 = 0) (1/2 = 1), gen(slept_under_anynet)
```

```
lab def slept_under_anynet 1 "Yes" 0 "No"
lab val slept_under_anynet slept_under_anynet

recode w109 (1/5 = 0)(6 = 1)(7 = 2)(8 = 3), gen (grouped_Rel)
label define grouped_Rel 0 "Christians" 1 "Muslim" 2 "No␣Religion" 3
    ↪  "Others"
lab val grouped_Rel grouped_Rel
gen Christians = .
replace Christians = 1 if grouped_Rel == 0
replace Christians = 0 if grouped_Rel == 1 | grouped_Rel == 2 |
    ↪ grouped_Rel == 3
gen Muslims = .
replace Muslims = 1 if grouped_Rel == 1
replace Muslims = 0 if grouped_Rel == 0 | grouped_Rel == 2 | grouped
    ↪ _Rel == 3
gen Other_religions = .
replace Other_religions = 1 if grouped_Rel == 3
replace Other_religions = 0 if grouped_Rel == 0 | grouped_Rel == 1 |
    ↪  grouped_Rel == 2
gen No_religion = .
replace No_religion = 1 if grouped_Rel == 2
replace No_religion = 0 if grouped_Rel == 0 | grouped_Rel == 1 |
    ↪ grouped_Rel == 3

recode wealth_cat (1/2 = 0)(3 = 1)(4/5 = 2), gen (gwealth_cat)
label define gwealth_cat 0 "Poor␣families" 1 "middle␣familes" 2 "
    ↪ Rich␣families"
lab val gwealth_cat gwealth_cat
gen poor_families = .
replace poor_families = 1 if gwealth_cat == 0
replace poor_families = 0 if gwealth_cat == 1 | gwealth_cat == 2
gen middle_families = .
replace middle_families = 1 if gwealth_cat == 1
replace middle_families = 0 if gwealth_cat == 0 | gwealth_cat == 2
gen rich_families = .
replace rich_families = 1 if gwealth_cat == 2
replace rich_families = 0 if gwealth_cat == 0 | gwealth_cat == 1

lab def region 0 "Northern" 1 "Central" 2 "Southern"
lab val region region
gen North = .
replace North = 1 if region == 0
replace North = 0 if region == 1 | region == 2
gen Central = .
replace Central = 1 if region == 1
replace Central = 0 if region == 0 | region == 2
gen South = .
replace South = 1 if region == 2
replace South = 0 if region == 0 | region == 1

rename w501 Exp_to_Mal_message
recode Exp_to_Mal_message (2 = 0) (1 = 1)
```

```
lab def Exp_to_Mal_message 0 "No" 1 "Yes"
lab val Exp_to_Mal_message Exp_to_Mal_message

recode residence (1 = 1) (2 = 0)
lab drop residence
lab def residence 0 "Rural" 1 "Urban"
lab val residence residence

recode w110 (1 = 0) (2 = 1) (3 = 2) (4 = 5) (7 = 5) (5 = 3) (6 = 5)
    ↪ (8 = 4) (9 = 5), gen(Tribes)
lab def Tribes 0 "Chewa" 1 "Tumbuka" 2 "Lomwe" 3 "Yao" 4 "Ngoni" 5 "
    ↪ Others"
lab val Tribes Tribes
gen Chewa = .
replace Chewa = 1 if Tribes == 0
replace Chewa = 0 if Tribes == 1 | Tribes == 2 | Tribes == 3 |
    ↪ Tribes == 4 | Tribes == 5
gen Tumbuka = .
replace Tumbuka = 1 if Tribes == 1
replace Tumbuka = 0 if Tribes == 0 | Tribes == 2 | Tribes == 3 |
    ↪ Tribes == 4 | Tribes == 5
gen Lomwe = .
replace Lomwe = 1 if Tribes == 2
replace Lomwe = 0 if Tribes == 0 | Tribes == 1 | Tribes == 3 |
    ↪ Tribes == 4 | Tribes == 5
gen Yao = .
replace Yao = 1 if Tribes == 3
replace Yao = 0 if Tribes == 0 | Tribes == 1 | Tribes == 2 | Tribes
    ↪ == 4 | Tribes == 5
gen Ngoni = .
replace Ngoni = 1 if Tribes == 4
replace Ngoni = 0 if Tribes == 0 | Tribes == 1 | Tribes == 2 |
    ↪ Tribes == 3 | Tribes == 5
gen Others = .
replace Others = 1 if Tribes == 5
replace Others = 0 if Tribes == 0 | Tribes == 1 | Tribes == 2 |
    ↪ Tribes == 3 | Tribes == 4

table1_mc, by(HCSB) vars(hh_sex cat \ Age_grps cat \ residence cat \
    ↪  region cat \ grouped_Rel cat \ Tribes cat \ Educ_Levels cat
    ↪ \ gwealth_cat cat \ Exp_to_Mal_messag cat\ slept_under_anynet
    ↪  cat \) total(after) saving("HCSB_2021.xlsx")

egen TAs = group(tacode)

xtmelogit HCSB i.hh_sex i.middle_women i.older_women i.Muslims i.
    ↪ Other_religions i.No_religion i.Tumbuka i.Lomwe i.Yao i.Ngoni
    ↪  i.Others i.Exp_to_Mal_messag i.Primary_sch i.Sec_and_above i
    ↪ .middle_families i.rich_families i.residence i.Central i.
    ↪ South i.slept_under_anynet || TAs:

save working_data_2021_070124.dta, replace
```

```
#B. Fitting a clustered mixed logistic model and computing
    ↪ univariate residuals

rm(list=ls())
library(foreign)
library(GGally)
library(lme4)
library(compiler)
library(parallel)
library(boot)
library(lattice)
library(car)
library(ggplot2)
library(reshape2)
library(nnet)
library(ggrepel)
library(dplyr)
library(data.table)
library(readstata13)

mydata = read.dta("C:/Users/User/Desktop/Health␣seeking␣behaviors␣
    ↪ paper/MICS2021III.dta",convert.factors=F)

tmo<- Sys.time()

for (k in 1:1)
       {

       grpSize <-data.frame(mydata %>% count(mydata$ta_name))$n

       model <- glmer(HSB ~ hh_sex + middle_women + older_women +
           ↪ Muslims + Other_religions + No_religion + Tumbuka +
           ↪ Lomwe + Yao + Ngoni + Others + Exp_to_Mal_message +
           ↪ Sec_and_above + Primary_sch + middle_families + rich_
           ↪ families + residence + Central + South + slept_under_
           ↪ anynet + (1 | ta_name), data = mydata, family =
           ↪ binomial, control = glmerControl(optimizer = "bobyqa")
           ↪ ,
              nAGQ = 10)
              for (j in 1:110)
              {
                     dt <- data.frame(cbind(newcluster=1:110))
                     dt2 <- as.data.frame(dt[rep(1:nrow(dt),grpSize
                         ↪ ),])
              }

       HSBData <- data.frame(cbind(mydata,dt2))

       HSBData$FittedVal = fitted(model)

       HSBData$Residj <- HSBData$HSB - HSBData$FittedVal
```

```
        HSBData$grandmean = setDT(HSBData)[,lapply(.SD,mean,na.rm=
            ↪ TRUE),.SDcols="Residj"]

        write.dta(HSBData, paste0("c:/Users/User/Desktop/MICSIImxd.
            ↪ dta"))
        }
        tm1<- Sys.time()
        tm1 - tmo

#C. Computing group outlier statistic

outliermat <- matrix(NA,nrow = 110,ncol =8)
outliermat <- data.frame(outliermat)
colnames(outliermat) <- c("ID","meanclustdev","wtngrpVar","grandavg"
    ↪ ,"btwngrpVar","ratiovar","sqrtratio","stdratio")
pb <- txtProgressBar(min=1,max=100,style = 3)
tmo<- Sys.time()
outliermat_all = matrix(NA,nrow = 110,ncol =8)
colnames(outliermat_all) <- c("ID","meanclustdev","wtngrpVar","
    ↪ grandavg","btwngrpVar","ratiovar","sqrtratio","stdratio")
        for(k in 1:1)
        {
         outliermat[,1]<- 1:110
         outliermat[,2] <- setDT(HSBData)[,lapply(.SD,mean,na.rm=
             ↪ TRUE),by=ta_name,.SDcols="Residj"][,2]
         outliermat[,3]<- setDT(HSBData)[,lapply(.SD,var,na.rm=TRUE)
             ↪ ,by=ta_name,.SDcols="Residj"][,2]
         outliermat[,4]<- setDT(HSBData)[,lapply(.SD,mean,na.rm=TRUE
             ↪ ),by=ta_name,.SDcols="grandmean"][,2]
         outliermat[,5] <- sum(grpSize*(outliermat$meanclustdev -
             ↪ outliermat$grandavg))^{2}/(110-1)
         outliermat[,6]<- outliermat$wtngrpVar/outliermat$btwngrpVar
         outliermat[,7] <- sqrt(outliermat$ratiovar)
         outliermat[,8]<- (outliermat$sqrtratio - mean(outliermat$
             ↪ sqrtratio))/sqrt(var(outliermat$sqrtratio))

                if (k==1) {outliermat_all = outliermat}
                else {outliermat_all = rbind.data.frame(outliermat_
                    ↪ all,outliermat)}

                setTxtProgressBar(pb,k)
        }
        write.dta(outliermat_all, paste0("c:/Users/User/Desktop/
            ↪ TArsd",110,".dta"))

        tm1<- Sys.time()
        tm1 - tmo

rm(list=ls())
library(foreign)
library(GGally)
```

```
library(lme4)
library(compiler)
library(parallel)
library(boot)
library(lattice)
library(car)
library(ggplot2)
library(reshape2)
library(nnet)
library(ggrepel)
library(dplyr)
library(data.table)
library(readstata13)

outlierdata = read.dta("C:/Users/User/Desktop/TArsd110.dta",convert.
    ↪ factors=F)

outliervge <- ggplot(outlierdata,aes(x=outlierdata$ID,y=outlierdata$
    ↪ ratiovar))+labs(x="TA_name",y="Outlier␣HSB␣for␣mixed␣logit␣
    ↪ model")+geom_text(aes(label = outlierdata$ta_name), size = 2.
    ↪ 8, vjust = 0.5)+ theme_test()+geom_hline(yintercept = c(9.327
    ↪ 458696074492),color=c('red')) #cutoffchosen at 95th
    ↪ percentile of the group outlier Residj

ggsave('test.tiff', outliervge, device = "tiff", units="in", width=6
    ↪ , height=4.5, pointsize=12,bg="white", dpi = 1200)
```
